# Veterinary Legislation

**Published:** 1890-02

**Authors:** 


### EDITORIAL.

### VETERINARY LEGISLATION.

*The Committee on Army Legislation*, of the U. S. Veterinary Medical Association, report that the following bill, introduced into the House of Representatives, at Washington, by General H. H. Bingham, of Pennsylvania, has been read twice, and referred to the *Committee on Military Affairs* :—

FIFTY-FIRST CONGRESS, FIRST SESSION. H. R. 3912.

A BILL TO PROVIDE FOR THE ORGANIZATION AND RATE OF PAY OF A VETERINARY CORPS OF THE UNITED STATES ARMY :—*Be it enacted by the Senate and House of Representatives of the United States of America in Congress assembled,* That there shall be, and hereby is, established, as a part of the United States Army, a Veterinary Corps, which shall consist of one Veterinary Surgeon-General, with the rank, pay and allowances of a major of cavalry, who shall be appointed by the President of the United States, by selection, with and by the consent of the Senate ; four veterinarians, with the rank, pay and allowances of captains of cavalry ; ten assistant veterinarians, with the rank, pay and allowances of first lieutenants of cavalry, and ten assistant veterinarians, with the rank, pay and allowances of second lieutenants of cavalry.

SEC. 2. That as soon as practicable after the passage of this act, the President of the United States shall appoint a Veterinary Medical Examining Board, which shall consist of the Veterinary Surgeon-General, two officers of cavalry and two officers of the Medical Department, whose duty it shall be to examine such candidates as shall present themselves for examination for appointment in the Veterinary Corps, and shall report and certify to the Secretary of War the names of the candidates who shall have passed the highest examination satisfactory to said Board.

SEC. 3. That upon the receipt from the said Examining Board of the certificates of the candidates who shall have passed the highest satisfactory examination, the President of the United States shall appoint to the various offices junior to the Veterinary Surgeon-General, the said appointees to take rank according to the order of merit certified by said Examining Board, not to exceed the number provided for in Section 1 of this act.

SEC. 4. That all veterinary surgeons of the United States Army who, at the passage of this act, shall be in service, may be granted three months' leave of absence with full pay, for the purpose of preparing themselves for examination.

SEC 5. That the Secretary of War shall hereafter appoint, from time to time, a Veterinary Examining Board, which shall consist of the Veterinary Surgeon-General and two veterinarians of the United States Army Veterinary Corps, to examine candidates for the position of assistant veterinarians, with the rank of second lieutenant and for promotion in the corps.

SEC. 6. That promotion below the rank of field officer shall be by seniority, but no officer of this corps shall be entitled to promotion thereby until he shall have been examined and approved by a veterinary examining board; and if any such officer fail on examination he shall be suspended from promotion for one year, when he shall be re-examined before a like board, and in case of failure on such re-examination he shall be discharged from the service.

The Committee of Military Affairs of the House of Representatives is composed of:

| | | |
|---|---|---|
| Messrs. | Cutcheon, . . . . . . . . . . . . . | Michigan, *Chairman.* |
| | Rockwell, . . . . . . . . . . . . . | Massachusetts. |
| | Osborne, . . . . . . . . . . . . . | Pennsylvania. |
| | Spooner, . . . . . . . . . . . . . | Rhode Island. |
| | Williams, . . . . . . . . . . . . . | Ohio. |
| | Lansing, . . . . . . . . . . . . . | New York. |
| | Snider, . . . . . . . . . . . . . | Minnesota. |
| | Kinsey, . . . . . . . . . . . . . | Missouri. |
| | Spinola, . . . . . . . . . . . . . | New York. |
| | Wheeler, . . . . . . . . . . . . . | Alabama. |
| | Lanham, . . . . . . . . . . . . . | Texas. |
| | Wise, . . . . . . . . . . . . . | Virginia. |
| | Robertson, . . . . . . . . . . . . . | Louisiana. |
| | Carey, . . . . . . . . . . . . . | Wyoming. |

The Committee of Military Affairs of the Senate is composed of:—

| | | |
|---|---|---|
| Messrs. | Hawley, . . . . . . . . . . . . . | Connecticut, *Chairman.* |
| | Cameron, . . . . . . . . . . . . . | Pennsylvania. |
| | Manderson, . . . . . . . . . . . . . | Nebraska. |
| | Stewart, . . . . . . . . . . . . . | Nevada. |
| | Davis, . . . . . . . . . . . . . | Minnesota. |
| | Cockrell, . . . . . . . . . . . . . | Missouri. |
| | Hampton, . . . . . . . . . . . . . | South Carolina. |
| | Walthall, . . . . . . . . . . . . . | Mississippi. |
| | Bate, . . . . . . . . . . . . . | Tennessee. |

Every member of the veterinary profession, and every friend of the advancement of veterinary medicine in the United States, should use his individual efforts, at once, to communicate with his Representative at Washington, and to see that the necessity for the passage of the bill is properly placed before Congress. The United States is the only civilized country in the world where the military veterinarian is not a commissioned officer. The annual loss, by death and disease, in the U. S. Army is greater than in the army of any other country. The small corps provided for in the above bill will undoubtedly save the government annually many times the cost of its support ; it will open to the veterinarians of the country a higher training school, of most interesting investigations, whose results will not only increase the effectiveness of the mounted service of the army, but will be of untold advantage to the agricultural community throughout the whole land.

Let every one see at once what he can do to favor the passage of this bill and let him report at once to either

DR. R. S. HUIDEKOPER, 111 South 20th St., Philadelphia ;

DR. D. LEMAY, Fort Riley, Kansas ;

DR. COOPER CURTICE, Depart. of Agriculture, Washington,

*Committee on Army Legislation, U. S. Vet. Med. Association.*

